# Moral identity internalization and symbolization in relation to body donation willingness: a cross-sectional study among 2,327 Chinese health professions students

**DOI:** 10.3389/fpubh.2026.1929251

**Published:** 2026-10-08

**Authors:** Liyuan Wang, Cheng Li, Peng Yu, Lei Zhang, Wenbo Du, Yuan Yuan

**Affiliations:** 1 School of Basic Medical Sciences, Bengbu Medical University, Bengbu, China; 2 School of Mental Health, Bengbu Medical University, Bengbu, China

**Keywords:** body donation willingness, China, health professions students, internalization, moral identity, symbolization

## Abstract

**Background:**

Body donation is essential to medical education, yet a global donor shortage persists. While prior research has identified demographic and attitudinal correlates of donation willingness, the deeper psychological factors associated with this decision remain largely unexplored. Moral identity—comprising internalization (private moral self-integration) and symbolization (public moral expression)—is a well-established prosocial motivator, but its role in body donation, a context defined by mortality salience and intangible outcomes, remains unexplored.

**Methods:**

In this cross-sectional study, 2,327 undergraduate health professions students from Bengbu Medical University, China, completed validated measures of moral identity, death anxiety, donation-related experiences, and willingness to donate their body. Multivariable logistic regression with progressive covariate adjustment estimated independent associations of internalization and symbolization with donation willingness, controlling for sociodemographic, educational, experiential, and death anxiety factors. Subgroup analyses examined effect heterogeneity.

**Results:**

Both dimensions were independently associated with donation willingness. In fully adjusted models, each standard-deviation increase in internalization was associated with 56.1% higher odds of willingness (OR = 1.561; 95% CI: 1.391–1.753), compared with 22.6% higher odds for symbolization (OR = 1.226; 95% CI: 1.094–1.372). Internalization showed a graded association with donation willingness (highest vs. lowest tertile: OR = 2.701; 95% CI: 2.040–3.575), whereas symbolization was significantly associated with donation willingness only in the highest tertile. Associations were observed across most subgroups, but were not significant among nursing students or religious participants.

**Conclusions:**

In this sample, moral identity internalization was more strongly associated with body donation willingness than symbolization. This suggests that internalized moral values may play a more important role than public moral expression in end-of-life donation decisions. The null findings in nursing students and religious participants suggest that the relevance of moral identity may vary across subgroups, though these exploratory findings require confirmation in future studies. This study introduces a perspective that complements the knowledge-attitude-practice paradigm, suggesting that moral identity may be a modifiable psychological construct worthy of further investigation in donation promotion efforts.

## Background

Body donation constitutes an indispensable cornerstone of anatomical education and scientific research within medical training. Yet, a persistent global shortage of donor bodies continues to impede the cultivation of medical professionals and the progress of biomedical research (1, 2). In response, existing studies have identified multiple factors associated with donation willingness, including demographic characteristics, procedural knowledge, specific attitudes toward death (e.g., death anxiety) (3, 4), and generalized altruistic dispositions (5). While this body of work offers a valuable preliminary framework, the deeper, more stable psychological architecture underlying such ultimate altruistic decisions remains largely unexplored. A critical gap persists in understanding how moral identity (6)—the degree to which individuals internalize moral traits as central to their self-concept—systematically shapes willingness regarding posthumous bodily disposition. Although moral identity is well-established as a core motivational source of prosocial behavior (7, 8), its distinctive role in the specific context of body donation—a context defined by high mortality salience, concerns about bodily integrity, and the deferred, intangible nature of outcomes—has not been previously examined.

Moral identity refers to the extent to which individuals psychologically internalize a set of moral traits (e.g., caring, fairness, and integrity) and the centrality of these traits to their self-concept. According to the influential theoretical framework proposed by Aquino and Reed (6), this construct comprises two interrelated yet functionally distinct dimensions: internalization, which reflects the intrinsic importance of moral traits to self-definition, and symbolization, which captures the motivation to express these traits to others through public actions. A substantial body of evidence has demonstrated that moral identity serves as a fundamental psychological resource driving prosocial behaviors ranging from everyday helping to significant self-sacrifice (9, 10). Nevertheless, extant research has largely treated moral identity as a unidimensional construct or focused on immediate, observable altruistic acts, thereby overlooking its potentially unique role in body donation decisions—decisions characterized by high mortality salience, bodily integrity concerns, and the deferred, intangible nature of outcomes (11). Body donation not only demands altruism that transcends the boundary of life but also directly confronts individuals with their own mortality. The psychological processes underlying this decision are therefore likely to differ substantively from those involved in other charitable behaviors (12). Theoretically, individuals high in internalization, owing to their highly integrated moral self-schema, may be more inclined to perceive donation as a natural extension of their intrinsic values—one that confers meaning upon the end of life. In contrast, individuals high in symbolization may be more motivated by social approval and impression management; yet this motivational pathway is likely attenuated in the context of posthumous donation, where outcomes remain unobservable to others (8, 13, 14). Although some studies have begun to explore the psychological mechanisms underlying body donation willingness (15), no study has yet tested whether internalization and symbolization are differentially associated with body donation willingness (5).

This gap originates, in part, from two fundamental limitations in the extant psychological literature on body donation. First, most studies have relied on demographic variables or attitudinal surveys, with limited attention to stable personality traits or deep-seated psychological structures, thereby constraining insight into the root motivational drivers of such behavior (16). This gap is compounded by a second limitation: even among the few studies that have ventured into psychological mechanisms, most have treated “altruism” as a monolithic construct, failing to disentangle the distinct and potentially competing psychological pathways that it subsumes (12). For instance, donation motivated by internal value alignment vs. donation driven by external social expectations may entail fundamentally different psychological dynamics and stability (17). This lack of theoretical granularity impedes the precise design of effective intervention strategies (5). Health professions students, as the future backbone of the healthcare workforce and potential advocates for donation, constitute an important population for investigating this issue (18). Although research on this key group has examined the influence of death education or cadaveric dissection experience (19, 20), a recent study of Chinese clinical medical students found that although 96% agreed with the value of donation, only 38.22% expressed willingness to donate their bodies, with traditional culture, family values, and religious factors playing important roles (21). Moreover, no study to date has systematically incorporated the dual-dimensional framework of moral identity into analysis while simultaneously controlling for confounders such as death anxiety and academic discipline.

To address these knowledge gaps, the present study conducted a large-scale cross-sectional survey among students enrolled at a medical university in China. For the first time in this research domain, we introduce the dual-dimensional Moral Identity Scale developed by Aquino and Reed (6), with the following core objectives: (1) to quantify the independent associations of the internalization and symbolization dimensions of moral identity with willingness to donate one's body; (2) to evaluate the robustness of these associations after adjusting for a multi-level set of confounders, including sociodemographic characteristics, death anxiety, medical education experiences (e.g., anatomy coursework, cadaveric dissection experience), and personal experiential factors; and (3) to explore, through subgroup analyses, the heterogeneity of these associations across key subgroups such as academic discipline (e.g., clinical medicine, nursing) and religious affiliation. We hypothesized that both dimensions of moral identity would be independently and positively associated with donation willingness, and that the association of internalization would be stronger than that of symbolization, given the inherently non-observable nature of outcomes in body donation decisions and the typically attenuated motivational influence of symbolization in contexts lacking social recognition (8). No a priori hypotheses were formulated for the exploratory subgroup analyses. This study offers a novel theoretical perspective and empirical evidence for understanding body donation at the level of moral self-identity.

## Methods

### Study design and data collection

This cross-sectional investigation took place at Bengbu Medical University in Anhui Province, China, from September through November 2025. Participants were recruited using convenience sampling from the current health professions student population. Inclusion criteria required respondents to: (1) be aged 18 years or older; (2) hold full-time undergraduate enrollment status; and (3) demonstrate the ability to understand the survey content and provide voluntary informed consent.

Questionnaires were distributed via Chaoxing Learning Pass (developed by Beijing Chaoxing Erya Education Technology Co., Ltd., www.chaoxing.com), the official learning management system universally used by all students at our institution for routine educational activities. The platform incorporated encryption protocols for data security, automatic prevention of duplicate entries via IP address tracking, and monitoring of response duration to identify irregular patterns. The initial questionnaire was released on September 10, 2025, and a single reminder was sent through the platform 2 weeks after the initial release. To further enhance response rates and minimize missing data, the research team coordinated with the Department of Teaching to arrange classroom reminders at two critical timepoints: (1) shortly after questionnaire release, during which instructors invited students in class to participate in the survey and encouraged their participation, and (2) approaching the survey deadline for a second reminder. These standardized classroom announcements were uniformly administered across courses through departmental teaching committees, with all instructors following identical scripts.

The questionnaire remained accessible for 2 months, from September 10, 2025, to November 10, 2025. Students were informed—both in the questionnaire interface and during classroom announcements—of the following: (1) the complete voluntary nature of participation; (2) the estimated completion time of approximately 10 mins; and (3) the final study deadline. On the first page of the questionnaire, respondents were informed of the study purpose, the voluntary nature of participation, and their right to withdraw at any time; completion and submission of the questionnaire constituted informed consent. No incentives were offered for participation.

A total of 2,500 electronic questionnaires were distributed to eligible undergraduate health professions students through the platform. We received 2,396 completed responses. All items in the online survey were designated as mandatory fields on the platform, which technically precluded submission of incomplete responses. As a result, no missing data occurred across any of the study variables. Participant anonymity was maintained throughout data collection and analysis. To ensure data quality, all questionnaires underwent logical verification and validation checks. Sixty-nine questionnaires exhibiting clear patterns of invalid responses (e.g., selecting identical options for all items) were excluded from the analysis. The final sample for data analysis comprised 2,327 valid questionnaires from students enrolled in clinical medicine, nursing, and related health science disciplines. The majority of participants (*n* = 2,243, 96.4%) were aged 18–21 years.

### Ethical considerations

Ethical approval was obtained from the University's Institutional Review Board (Approval No. 2025-591). Prior to participation, all respondents were briefed on the research objectives and provided electronic informed consent.

### Sociodemographic and experiential characteristics

Information on sociodemographic and experiential characteristics was collected using structured survey items with predefined response categories, including age (continuous variable in years), gender (male/female), major (clinical medicine/nursing/other health sciences), anatomy course enrollment (yes/no), cadaver exposure (yes/no), religious belief (yes/no), family member in medical field (yes/no), knowledge of body donation, hometown location (urban/suburban/rural), and bereavement experience (yes/no).

In this sample, religious belief was assessed as a binary variable (yes/no) due to the small number of participants endorsing specific traditions. Accordingly, subgroup analyses by specific religious denomination were not feasible.

Self-rated knowledge of body donation was assessed on a four-category scale (excellent/good/fair/poor or none), with higher categories indicating greater perceived knowledge about body donation for medical education and research.

### Death anxiety

Death anxiety was assessed using the Chinese version of Templer's Death Anxiety Scale (T-DAS), which was previously validated by Yang et al. (22). No new translation was performed by the present authors. This instrument comprises 15 binary-response items (yes = 1, no = 0), yielding total scores ranging from 0 to 15, with higher scores indicating greater death anxiety. The original English version demonstrated satisfactory reliability (KR-20 = 0.76) (3). Yang et al. (22) developed the Chinese adaptation through bidirectional translation, expert consultation, and preliminary testing, reporting adequate internal consistency among a sample of 1,118 Chinese university students and hospice care providers (α = 0.71). In the present sample, Cronbach's α was 0.75, indicating acceptable reliability.

### Moral identity

Moral identity was measured using Aquino and Reed's (6) Moral Identity Scale (MIS), a 10-item instrument comprising two dimensions: internalization (five items, reflecting the centrality of moral traits to self-concept) and symbolization (five items, capturing outward manifestations of moral traits). Respondents rated each item on a five-point Likert scale from 1 (strongly disagree) to 5 (strongly agree), with total scores ranging from 10 to 50 and higher scores reflecting stronger moral self-identification. The original scale demonstrated satisfactory reliability across five validation samples (internalization: α = 0.73–0.83; symbolization: α = 0.71–0.85). Because no previously validated Chinese version of the MIS was available, we developed a Chinese adaptation following standard translation-back-translation procedures: two bilingual translators independently produced Chinese versions, followed by back-translation by four blinded reviewers with expertise in medicine and psychology, and semantic equivalence was verified through expert consensus. The resulting Chinese adaptation exhibited acceptable internal consistency in our sample (internalization: α = 0.76; symbolization: α = 0.84).

### Body donation willingness

Body donation willingness was assessed using a single dichotomous question: “Would you be willing to donate your body for medical education and scientific research after death?” Responses were coded as 0 (no) or 1 (yes).

### Statistical analysis

Scale reliability was evaluated using Cronbach's α. Descriptive statistics were used to summarize participant characteristics. Categorical variables were presented as frequencies and percentages, while continuous variables were expressed as mean ± standard deviation (SD). Between-group comparisons (willing vs. unwilling) were performed using chi-square tests for categorical variables and independent samples t-tests for continuous variables.

Multivariable logistic regression models with progressive adjustments were employed to assess the associations of Moral Identity Scale internalization (MIS-Internalization) and symbolization (MIS-Symbolization) with body donation willingness. Three progressively adjusted models were constructed: Model I (unadjusted); Model II (adjusted for gender, age, academic program, cadaveric dissection experience, religious affiliation, prior exposure to cadaveric specimens, personal bereavement experience, self-rated knowledge of body donation, family members in healthcare, and hometown location); Model III further adjusted for the other moral identity dimension (internalization adjusted for symbolization and vice versa).

Moral identity scores were analyzed in three forms: (1) as continuous variables; (2) as z-standardized scores (mean = 0, SD = 1) to express effect sizes as odds ratios per 1-SD increase; and (3) as tertile categories (T1: lowest, T2: middle, and T3: highest) to examine graded associations. Tests for linear trend across tertiles were conducted by modeling the tertile categories as ordinal variables in the regression models.

To examine whether the associations between moral identity and body donation willingness varied across subgroups, we conducted formal interaction tests. For each subgroup variable (gender, anatomy course enrollment, medical program, bereavement experience, cadaver exposure, knowledge of body donation, and religious belief), interaction terms were created by multiplying the continuous MIS subscale scores by the dichotomous or categorical subgroup indicator variable. These interaction terms were then included in the logistic regression model alongside the main effects for the independent variable and subgroup variable. The *P*_interaction_ value was derived from the coefficient of the interaction term in the regression model, with *P*_interaction_ < 0.05 indicating a statistically significant interaction. A significant interaction would suggest that the strength or direction of the association differs across subgroups.

Statistical analyses were performed using SPSS version 26.0 (IBM Corp., Armonk, NY, USA) and EmpowerStats version 5.2 (X&Y Solutions, Inc., Boston, MA, USA). Statistical significance was set at a two-tailed α = 0.05.

## Results

### Participant characteristics by body donation willingness

Table 1 presents the demographic, educational, and experiential characteristics of participants stratified by donation willingness. Among the 2,327 health professions students surveyed, 560 (24.06%) expressed willingness to donate their bodies for medical education or research.

**Table 1:** Participant characteristics by body donation willingness.

Characteristics	Overall	Donation willingness		*P*-value
		Unwilling	Willing	
	*N* = 2,327	*N* = 1,767	*N* = 560	
**Gender**				0.225
Male	957 (41.13%)	739 (41.82%)	218 (38.93%)	
Female	1,370 (58.87%)	1,028 (58.18%)	342 (61.07%)	
**Age**	18.73 ± 1.00	18.73 ± 1.04	18.73 ± 0.86	0.920
**Major**				<0.001
Clinical Medicine	1,523 (65.45%)	1,116 (63.16%)	407 (72.68%)	
Nursing	387 (16.63%)	307 (17.37%)	80 (14.29%)	
Other Health Sciences	417 (17.92%)	344 (19.47%)	73 (13.04%)	
**Anatomy course enrollment**				0.070
Yes	1,770 (76.06%)	1,360 (76.97%)	410 (73.21%)	
No	557 (23.94%)	407 (23.03%)	150 (26.79%)	
**Religious belief**				0.005
Yes	93 (4.00%)	82 (4.64%)	11 (1.96%)	
No	2,234 (96.00%)	1,685 (95.36%)	549 (98.04%)	
**Cadaver exposure**				0.161
Yes	1,711 (73.53%)	1,312 (74.25%)	399 (71.25%)	
No	616 (26.47%)	455 (25.75%)	161 (28.75%)	
**Family member in medical field**				0.001
Yes	1,117 (48.00%)	815 (46.12%)	302 (53.93%)	
No	1,210 (52.00%)	952 (53.88%)	258 (46.07%)	
**Knowledge of body donation**				<0.001
Excellent	95 (4.08%)	66 (3.74%)	29 (5.18%)	
Good	573 (24.62%)	400 (22.64%)	173 (30.89%)	
Fair	1,089 (46.80%)	830 (46.97%)	259 (46.25%)	
Poor/none	570 (24.50%)	471 (26.66%)	99 (17.68%)	
**Hometown location**				0.135
Urban	627 (26.94%)	458 (25.92%)	169 (30.18%)	
Suburban	611 (26.26%)	468 (26.49%)	143 (25.54%)	
Rural	1,089 (46.80%)	841 (47.59%)	248 (44.29%)	
**Bereavement experience**				0.628
Yes	1,501 (64.50%)	1,135 (64.23%)	366 (65.36%)	
No	826 (35.50%)	632 (35.77%)	194 (34.64%)	
**T-DAS score**	7.29 ± 3.20	7.47 ± 3.21	6.72 ± 3.10	<0.001
**MIS-Internalization**	19.89 ± 3.18	19.51 ± 3.16	21.11 ± 2.92	<0.001
**MIS-Symbolization**	15.94 ± 3.42	15.61 ± 3.32	17.01 ± 3.52	<0.001

Values are presented as n (%) for categorical variables and mean ± SD for continuous variables. P-values were derived from chi-square tests for categorical variables and independent samples t-tests for continuous variables, comparing characteristics between participants willing and unwilling to donate their bodies. n, number of participants; SD, standard deviation; P, P-value; T-DAS, Templer's Death Anxiety Scale; MIS, Moral Identity Scale.

Participants willing and unwilling to donate their bodies showed no significant differences in gender (*P* = 0.225), age (18.73 ± 0.86 vs. 18.73 ± 1.04 years, *P* = 0.92), anatomy course enrollment (*P* = 0.070), cadaver exposure (*P* = 0.161), hometown location (*P* = 0.135), and bereavement experience (*P* = 0.628).

However, several characteristics differed significantly between groups. Clinical medicine students comprised a higher proportion of the willing group (72.68% vs. 63.16%, *P* < 0.001). Religious belief was less prevalent among willing participants (1.96% vs. 4.64%, *P* = 0.005), and students with family members in the medical field showed greater willingness to donate (53.93% vs. 46.12%, *P* = 0.001). Knowledge of body donation was also significantly associated with willingness (*P* < 0.001): those reporting good knowledge were more prevalent in the willing group (30.89% vs. 22.64%), whereas those reporting poor or no knowledge were less prevalent (17.68% vs. 26.66%).

Psychological and moral dimensions also differed markedly between groups. Willing group reported significantly lower death anxiety scores (T-DAS: 6.72 ± 3.10 vs. 7.47 ± 3.21, *P* < 0.001). Both dimensions of moral identity were substantially higher in the willing group: MIS-Internalization (21.11 ± 2.92 vs. 19.51 ± 3.16, *P* < 0.001) and MIS-Symbolization (17.01 ± 3.52 vs. 15.61 ± 3.32, *P* < 0.001).

### Association between MIS-internalization and body donation willingness

Table 2 presents the associations between MIS-Internalization and body donation willingness across three progressively adjusted logistic regression models. Statistically significant positive associations between MIS-Internalization and body donation willingness were observed across all models after sequential adjustment for potential confounders.

**Table 2:** Association between MIS-internalization and body donation willingness.

MIS-Internalization	Model I	Model II	Model III
	OR (95% CI) *P*-value	OR (95% CI) *P*-value	OR (95% CI) *P*-value
Donation willingness			
Z-MIS-Internalization	1.739 (1.563, 1.934) <0.001	1.686 (1.513, 1.880) <0.001	1.561 (1.391, 1.753) <0.001
MIS-Internalization	1.190 (1.151, 1.231) <0.001	1.179 (1.139, 1.220) <0.001	1.150 (1.109, 1.193) <0.001
MIS-Internalization Tertile			
T1	Reference	Reference	Reference
T2	1.953 (1.493, 2.555) <0.001	1.902 (1.445, 2.503) <0.001	1.732 (1.311, 2.288) 0.001
T3	3.488 (2.688, 4.527) <0.001	3.266 (2.501, 4.266) <0.001	2.701 (2.040, 3.575) <0.001
*P* for trend	<0.001	<0.001	<0.001

Z-MIS-Internalization was z-standardized (mean = 0, SD = 1). OR, odds ratio; CI, confidence interval. Model I: unadjusted. Model II: adjusted for gender, age, medical program, anatomy course enrollment, religious belief, cadaver exposure, bereavement experience, knowledge of body donation, family member in medical field, and hometown location. Model III: further adjusted for MIS-Symbolization.

In the unadjusted Model I, each standard deviation increase in standardized MIS-Internalization was associated with a 73.9% increase in the odds of body donation willingness (OR = 1.739, 95% CI: 1.563–1.934, and *P* < 0.001). The highest tertile (T3) showed 248.8% higher odds compared to the lowest tertile (T1) (OR = 3.488, 95% CI: 2.688–4.527, and *P* < 0.001).

After adjusting for sociodemographic characteristics, death anxiety, and body donation-related experiences in Model II, standardized MIS-Internalization remained associated with body donation willingness (OR = 1.686, 95% CI: 1.513–1.880, and *P* < 0.001), with T3 participants exhibiting 226.6% higher odds than T1 participants (OR = 3.266, 95% CI: 2.501–4.266, and *P* < 0.001).

In the fully adjusted Model III, with further adjustment for MIS-Symbolization, standardized MIS-Internalization remained associated with body donation willingness. Each standard deviation increase in MIS-Internalization was associated with a 56.1% increase in the odds of body donation willingness (OR = 1.561, 95% CI: 1.391–1.753, and *P* < 0.001). When analyzed using raw scores, each one-unit increase was associated with a 15.0% increase in odds (OR = 1.150, 95% CI: 1.109–1.193, and *P* < 0.001), providing a concrete metric for score interpretation. Tertile-based analysis showed the graded pattern across categories of MIS-Internalization. Compared to the lowest tertile (T1), participants in the middle tertile (T2) had 73.2% higher odds of body donation willingness (OR = 1.732, 95% CI: 1.311–2.288, and *P* = 0.001), while those in the highest tertile (T3) had 170.1% higher odds (OR = 2.701, 95% CI: 2.040–3.575, and *P* < 0.001). The linear trend test was statistically significant across tertiles (*P* for trend <0.001).

### Association between MIS-symbolization and body donation willingness

Table 3 presents the associations between MIS-Symbolization and body donation willingness across three progressively adjusted models. Significant positive associations were observed. The odds ratios decreased from Model II to Model III after adjustment for MIS-Internalization.

**Table 3:** Association between MIS-symbolization and body donation willingness.

MIS-Symbolization	Model I	Model II	Model III
	OR (95% CI) *P*-value	OR (95% CI) *P*-value	OR (95% CI) *P*-value
Donation willingness			
**Z-MIS-Symbolization**	1.545 (1.394, 1.712) <0.001	1.458 (1.311, 1.621) <0.001	1.226 (1.094, 1.372) <0.001
**MIS-Symbolization**	1.136 (1.102, 1.170) <0.001	1.116 (1.082, 1.152) <0.001	1.061 (1.027, 1.097) <0.001
MIS-Symbolization Tertile			
T1	Reference	Reference	Reference
T2	0.892 (0.675, 1.178) 0.420	0.880 (0.663, 1.169) 0.377	0.904 (0.676, 1.210) 0.498
T3	2.222 (1.740, 2.837) <0.001	1.969 (1.527, 2.538) <0.001	1.442 (1.103, 1.885) 0.007
*P* for trend	<0.001	<0.001	0.003

Z-MIS-Symbolization was z-standardized (mean = 0, SD = 1). OR, odds ratio; CI, confidence interval. Model I: unadjusted. Model II: adjusted for gender, age, medical program, anatomy course enrollment, religious belief, cadaver exposure, bereavement experience, knowledge of body donation, family member in medical field, and hometown location. Model III: further adjusted for MIS—Internalization.

In the unadjusted Model I, standardized MIS-Symbolization showed a significant association, with each standard deviation increase corresponding to a 54.5% increase in donation odds (OR = 1.545, 95% CI: 1.394–1.712, and *P* < 0.001). The highest tertile (T3) showed 122.2% higher odds compared to the lowest tertile (T1) (OR = 2.222, 95% CI: 1.740–2.837, and *P* < 0.001). After adjusting for sociodemographic characteristics, death anxiety, and body donation-related experiences in Model II, the standardized effect remained significant (OR = 1.458, 95% CI: 1.311–1.621, and *P* < 0.001), with T3 participants exhibiting 96.9% higher odds than T1 participants (OR = 1.969, 95% CI: 1.527–2.538, and *P* < 0.001).

In the fully adjusted Model III, which additionally controlled for MIS-Internalization, MIS-Symbolization was statistically significantly associated with body donation willingness. For standardized analysis, each standard deviation increase in MIS-Symbolization corresponded to a 22.6% increase in odds (OR = 1.226, 95% CI: 1.094–1.372, and *P* < 0.001). When analyzed using raw scores, each one-unit increase in MIS-Symbolization was associated with a 6.1% increase in odds (OR = 1.061, 95% CI: 1.027–1.097, and *P* < 0.001).

For MIS-Symbolization in Model III, participants in the middle tertile (T2) showed no statistically significant association with body donation willingness (OR = 0.904, 95% CI: 0.676–1.210, and *P* = 0.498). In contrast, those in the highest tertile (T3) demonstrated 44.2% higher odds associated with body donation willingness (OR = 1.442, 95% CI: 1.103–1.885, and *P* = 0.007). Despite this differential pattern across tertiles, the overall linear trend remained statistically significant (*P* for trend = 0.003).

### Consistency and variation of associations across subgroups

Stratified logistic regression analyses with interaction testing showed positive associations between moral identity dimensions and body donation willingness in most demographic and educational subgroups (Table 4). Significant effect modification was observed for medical program type in both dimensions, whereas cadaver exposure showed significant modification only for internalization. Other stratification variables (gender, anatomy course enrollment, bereavement experience, knowledge level, and religious belief) showed similar associations without significant effect modification.

**Table 4:** Association between moral identity and body donation willingness: subgroup analysis.

Donation willingness	MIS-Internalization	*P*_interaction_	MIS-Symbolization	*P*_interaction_
	OR (95% CI) *P*-value		OR (95% CI) *P*-value	
**Gender**		0.334		0.262
Male	1.157 (1.100, 1.217) <0.001		1.097 (1.050, 1.146) <0.001	
Female	1.197 (1.142, 1.254) <0.001		1.135 (1.088, 1.185) <0.001	
**Anatomy course enrollment**		0.315		0.962
Yes	1.191 (1.145, 1.239) <0.001		1.118 (1.079, 1.159) <0.001	
No	1.144 (1.068, 1.224) 0.001		1.116 (1.049, 1.188) <0.001	
**Medical program**		<0.001		0.027
Clinical Medicine	1.203 (1.155, 1.254) <0.001		1.119 (1.080, 1.160) <0.001	
Nursing	0.997 (0.914, 1.087) 0.942		1.028 (0.943, 1.120) 0.531	
Other Health Sciences	1.266 (1.148, 1.397) <0.001		1.226 (1.112, 1.352) <0.001	
**Bereavement experience**		0.426		0.324
Yes	1.167 (1.119, 1.217) <0.001		1.104 (1.063, 1.147) <0.0001	
No	1.202 (1.133, 1.274) <0.001		1.141 (1.082, 1.203) <0.0001	
**Cadaver exposure**		0.032		0.869
Yes	1.207 (1.158, 1.257) <0.001		1.115 (1.075, 1.156) <0.001	
No	1.111 (1.044, 1.183) <0.001		1.121 (1.056, 1.190) <0.001	
**Knowledge of body donation**		0.764		0.635
Excellent	1.092 (0.943, 1.266) 0.239		1.285 (1.075, 1.536) 0.006	
Good	1.194 (1.117, 1.276) <0.001		1.133 (1.067, 1.204) <0.001	
Fair	1.186 (1.126, 1.249) <0.001		1.109 (1.058, 1.163) <0.001	
Poor/none	1.183 (1.093, 1.281) <0.001		1.103 (1.030, 1.181) 0.005	
**Religious belief**		0.239		0.096
Yes	1.018 (0.791, 1.312) 0.888		0.871 (0.659, 1.151) 0.331	
No	1.182 (1.142, 1.224) <0.001		1.120 (1.086, 1.156) <0.001	

OR, odds ratio; CI, confidence interval; MIS, Moral Identity Scale. P interaction tests for heterogeneity of associations across subgroups. All models were adjusted for gender, age, medical program, anatomy course enrollment, religious belief, cadaver exposure, bereavement experience, knowledge of body donation, family member in medical field, and hometown location. Stratification variables were excluded from adjustment in their respective subgroup analyses.

The internalization dimension showed positive associations in most subgroups, with notable effect modification by medical program type (*P*_interaction_ <0.001). Clinical Medicine and Other Health Sciences students showed significant associations with donation willingness, whereas Nursing students did not. Cadaver exposure also showed significant effect modification (*P*_interaction_ = 0.032), with students having exposure experience showing larger odds ratios than those without. Significant associations were observed among those without religious beliefs, whereas no significant associations were found among those with religious beliefs. No significant effect modification was observed for gender, anatomy course enrollment, bereavement experience, or religious belief status.

The symbolization dimension showed similar positive associations in most subgroups, with significant effect modification by medical program type (*P*_interaction_ = 0.027). Clinical Medicine and Other Health Sciences students showed associations with donation willingness, whereas Nursing students showed no significant association. No significant effect modification was detected for gender, anatomy course enrollment, bereavement experience, cadaver exposure, or knowledge level. Similar to the internalization dimension, significant associations were observed among those without religious beliefs, whereas no significant associations were found among those with religious beliefs.

## Discussion

This cross-sectional study of 2,327 Chinese health professions students offers preliminary evidence on the dual-dimensional structure of moral identity in relation to body donation. Both internalization and symbolization were independently and positively associated with donation willingness. Internalization showed a graded association across tertiles, with participants in the highest tertile showing 170% higher odds of willingness than those in the lowest tertile (OR = 2.701), whereas the association for symbolization was significant only in the highest tertile. Notably, these associations were not significant in two subgroups: nursing students and participants with religious beliefs. These null findings suggest that the association between moral identity and donation willingness may be context-dependent.

The stronger association for internalization was observed within the “social recognition vacuum” that characterizes body donation decisions. While prior research has frequently juxtaposed these two dimensions (6, 23), it has seldom interrogated the conditions under which internalization outweighs symbolization. Body donation involves invisibility of outcomes, deferral of returns, and irrevocability of the act-characteristics that may minimize external social rewards (24). In this setting, the impression management and public performance on which symbolization depends are stripped away, whereas the drive for moral self-consistency that animates internalization becomes increasingly salient due to the heightened mortality salience inherent in the donation decision (25). This finding resonates with self-determination theory (26): when external reward contingencies are absent, integrated regulation (actions fully congruent with the self) becomes motivationally superior, whereas introjected regulation (serving self-esteem or social approval) rapidly attenuates. Viewed through the lens of terror management theory (27, 28), internalization could be understood not only as a moral disposition but also as an existential resource. Recent experimental research further confirms that mortality salience can directly enhance individuals' charitable donation intentions, an effect that remains robust after controlling for income and socioeconomic status (29). It may transform corporeal donation into a contribution to medical education, potentially enabling individuals to achieve symbolic meaning beyond their lifetime, a process that qualitative evidence suggests donation volunteers conceptualize as “life extension” and “self-transcendence” (30). Previous research indicates that the internalization dimension supports the persistence of prosocial behavior by providing an intrinsic meaning system, whereas the symbolization dimension lacks this self-sustaining function because its rewards depend on social recognition and others' attention (8, 31). In the postmortem act of body donation, the absence of external recognition may weaken the motivational effect of symbolization. The present study found a stronger association for internalization than for symbolization, which is consistent with this proposed mechanism. Accordingly, we speculate that in prosocial decisions characterized by high mortality salience and low behavioral visibility, internalization may serve as both a motivational source and a meaning-making resource. This interpretation, however, requires further empirical testing.

The null association among nursing students represents a notable boundary condition for the motivational relevance of moral identity in this context. One speculative interpretation is that nursing education cultivates an ethics of care that is context-bound and oriented toward living patients (32, 33), which may limit the transfer of moral self-perceptions to posthumous donation decisions. We term this the professional–self schema compartmentalization hypothesis. Nursing students' clinical training is structured around the “responsive other”—the suffering, vulnerable, and comfort-seeking patient (34, 35). However, recent scoping review evidence suggests an interesting tension: nursing students' cadaveric dissection experiences can, in fact, construct “an image of care for the patient before death,” foster understanding of the meaning of existence after death, and promote professional identity development (36). This suggests that nursing students are capable of establishing meaningful connections with the cadaver; however, current dissection pedagogy may not anchor this meaning-making within the narrative framework of nursing ethics. Moral identity, as a generalized psychological resource, may encounter difficulty transferring across domains to activate donation decisions (23). This interpretation is indirectly supported by the observation that nursing students in our sample did not differ from other majors in moral identity levels, yet showed no association between moral identity and donation willingness. This disruption may not signify an absence of moral motivation but rather could reflect a restructuring of moral self-functioning through professional socialization (37). If this interpretation is correct, the target of intervention for nursing students would not be to “enhance moral identity” *per se* but to reconstruct the moral meaning of the cadaver within nursing's professional schema—reanchoring dissection pedagogy within the core narrative of nursing ethics: the cadaver as a “former patient” (38), dissection as the final enactment of a patient's end-of-life wishes (39), and donation as an extension of nursing care (40). This hypothesis, however, requires direct empirical testing.

The null association among religious participants presents an equally compelling, yet symmetrically opposite, boundary condition. Whereas, nursing students possess abundant moral resources that fail to transfer, religious participants—despite holding worldviews explicitly equipped to confer meaning upon death—demonstrate no translation of moral identity into donation willingness. This finding qualifies the assumption that religious meaning frameworks universally facilitate prosocial behavior in end-of-life contexts (41, 42). We speculate that religious doctrines that sacralize the body may render posthumous donation cognitively dissonant, even for individuals with strong moral self-concepts (43)—a possibility we term the sacred boundary preservation hypothesis. Within these interpretive frameworks, bodily integrity is not merely a biological preference but a theological imperative; donation, however altruistically motivated, risks being construed as desecration rather than gift (44). This interpretation is indirectly supported by recent cross-religious survey evidence: although Christians demonstrated higher awareness of organ donation, their actual registration rates and consent rates for uterine donation were lower; Muslim families showed significant reservations regarding donation requests when the deceased's wishes were unknown (45). Consistent with this interpretation, symbolization showed the most pronounced attenuation among religious participants (OR = 0.871). If religious individuals were simply “less altruistic,” both moral identity dimensions would be equally suppressed. Instead, the selective erosion of symbolization suggests that what is inhibited is not moral selfhood *per se*, but its public, behavioral expression in a domain perceived as theologically sensitive. If this interpretation is correct, promotional strategies for religious populations might shift from emphasizing “moral duty” to seeking “theological alignment” (45). This remains speculative without data on specific religious denominations.

The juxtaposition of these two null effects—nursing students (moral resources intact, translation blocked) and religious participants (meaning frameworks intact, moral self-disengaged)—suggests a deeper theoretical architecture. As a theoretical framework for future research, we propose the moral self-meaning construction model, which posits that altruistic behavior may arise from the interplay of three components: (1) motivational resources (moral identity internalization and symbolization); (2) situational activation conditions (mortality salience, outcome visibility); and (3) meaning-making frameworks that bridge the self and the act (professional ethics, religious worldviews, and scientific cognition). Internalization operates as a cross-situationally robust motivational resource, yet its translational efficacy is contingent upon the accessibility of meaning frameworks that can assimilate donation into the individual's existential or professional narrative. Symbolization, by contrast, is a context-dependent motivational resource that exerts influence only in settings where social recognition is salient (8). When meaning frameworks are structurally misaligned with the core narratives of moral identity—whether through schematic compartmentalization (nursing) or sacred boundary preservation (religion)—moral identity may not be translated into behavioral intention. This model is intended to provide a conceptual framework for understanding the conditions under which moral identity may or may not translate into donation intentions.

This model also illuminates a striking asymmetry in our findings: whereas religious participants showed complete decoupling, non-religious participants—who constituted 96% of the sample—exhibited the strongest translation of moral identity into donation willingness. This pattern is consistent with the proposed model. Non-religious individuals, unconstrained by theological prohibitions on bodily integrity, and lacking alternative transcendental meaning resources, may rely more heavily on moral self-consistency as a source of existential meaning in the face of mortality. Their moral identity is not merely a prosocial disposition but a secular answer to the question of what endures after death. This interpretation is further supported by the gradient pattern observed for symbolization across knowledge levels: among participants with excellent donation knowledge, the association for symbolization was amplified (OR = 1.285). For non-religious individuals, cognitive involvement (deep understanding of donation procedures, scientific value, and societal benefit) may partially substitute for, or synergize with, value involvement (moral self-consistency) (23). They do not require divine sanction; they require evidence that their act will matter within an immanent, secular chain of consequence. If this interpretation holds, promotional practices might consider reframing body donation not exclusively as an altruistic moral act, but also as participation in a meaningful scientific legacy (38).

Several limitations of this study warrant careful consideration. Previous meta-analytic evidence suggests that personality traits show only small associations with body donation, highlighting the need to identify more proximal psychological factors such as moral identity (46). First, regarding sample characteristics and generalizability: our participants represented a relatively homogeneous demographic confined to undergraduate health sciences students (majority aged 18–21 years) from a single institution in China, recruited via convenience sampling. This narrow demographic profile significantly limits the generalizability of our findings. At this developmental stage, moral identity—comprising both internalization and symbolization dimensions—remains in an active phase of formation and reorganization, which may not reflect the stability and coherence of these constructs in mature adult populations. Critically, the convenience sampling methodology inherently limits representativeness beyond the specific institutional and educational context studied. Most importantly, our sample composition does not reflect the diversity of actual posthumous donors in real-world settings, who span diverse ages, educational backgrounds, occupations, cultural contexts, and socioeconomic statuses. The psychological mechanisms driving donation willingness in our young, health-sciences-focused sample may differ substantially from those operating in older, broader populations facing actual end-of-life decisions.

Second, regarding measurement: the use of a single dichotomous item to assess donation willingness, while pragmatically efficient, fails to capture the full multi-stage trajectory from intention to actual behavioral manifestation. Subsequent research should incorporate behavioral endpoints such as registered donor status and documented family communication, which would provide more robust evidence of the intention–behavior relationship.

Third, regarding mechanistic understanding: our findings revealed notable null effects among nursing students and religious participants that warrant deeper investigation. These differential patterns suggest potential schema compartmentalization and sacred boundary preservation mechanisms. Future qualitative inquiry is needed to substantiate these proposed mechanisms and provide context-sensitive knowledge for intervention design.

Fourth, regarding causality and external validity: the single-center, cross-sectional design inherently precludes causal inference and limits generalizability beyond this specific educational and cultural context. Multicenter longitudinal studies and experimental interventions are needed to establish directionality, test the plasticity of these psychological constructs, and validate generalizability across diverse demographic contexts.

Notwithstanding these limitations, this study provides initial empirical evidence that moral identity internalization is associated with body donation willingness in this sample, and offers a theoretical framework for understanding the conditions under which this association may vary. The null findings among nursing students and religious participants suggest that not all individuals respond to the same discourse, and that effective promotion may need to be tailored to different meaning frameworks. These findings suggest that body donation promotion may benefit from moving beyond information provision toward meaning co-construction with target audiences. This perspective does not negate the role of moral identity but acknowledges that multiple pathways may exist for different populations. These conclusions, however, are speculative and require further research.

## Conclusions

This study provides the first empirical application of the dual-dimensional model of moral identity to body donation. Internalization was more strongly associated with donation willingness than symbolization (per-SD increase: 56.1% vs. 22.6%), but these associations were absent in nursing students and religious participants, suggesting that professional and existential meaning systems may moderate this link. By complementing knowledge-deficit frameworks with identity-based mechanisms, these findings suggest that moral identity may be a potentially modifiable construct worthy of further intervention research. However, causal inferences are precluded by the single-center, cross-sectional design and the predominantly young, homogeneous sample; interaction tests were conducted but require confirmation in larger, more diverse samples. Future research should employ longitudinal and experimental designs with more diverse populations and incorporate fine-grained measures of religious affiliation.

## Data Availability

The raw data supporting the conclusions of this article will be made available by the authors, without undue reservation.

## References

[1] Puci MV Sotgiu MA Muresu N Saderi L Moretti V Ratti S . Body donation under Italy's recent legal reforms: a cross-sectional study of attitudes, beliefs, and educational gaps among medical students and faculty. Anat Sci Educ. (2025) 18:923–36. doi: 10.1002/ase.70084 40619569 PMC12413481

[2] Wang Y Dong P Zhou Y Gao S Zhang L. Motivational factors affecting body and organ donation in China identified based on grounded theory. Transplant Proc. (2024) 56:2115–23. doi: 10.1016/j.transproceed.2024.11.005 39603965

[3] Templer DI. The construction and validation of a Death Anxiety Scale. J Gen Psychol. (1970) 82:165–77. doi: 10.1080/00221309.1970.9920634 4394812

[4] Cai W Tang YL Wu S Li H. Scale of Death Anxiety (SDA): development and validation. Front Psychol. (2017) 8:858. doi: 10.3389/fpsyg.2017.00858 28620327 PMC5449485

[5] Post SG. Altuism, happiness, and health: it‘s good to be good. Int J Behav Med. (2005) 12:66–77. doi: 10.1207/s15327558ijbm1202_4 15901215

[6] Aquino K Reed A II. The self-importance of moral identity. J Pers Soc Psychol. (2002) 83:1423–40. doi: 10.1037/0022-3514.83.6.1423 12500822

[7] Krettenauer T Lefebvre JP Hardy SA Zhang Z Cazzell AR. Daily moral identity: linkages with integrity and compassion. J Pers. (2022) 90:663–74. doi: 10.1111/jopy.12689 34773252

[8] Winterich KP Aquino K Mittal V Swartz R. When moral identity symbolization motivates prosocial behavior: the role of recognition and moral identity internalization. J Appl Psychol. (2013) 98:759–70. doi: 10.1037/a0033177 23751218

[9] Reed A II Aquino KF. Moral identity and the expanding circle of moral regard toward out-groups. J Pers Soc Psychol. (2003) 84:1270–86. doi: 10.1037/0022-3514.84.6.1270 12793589

[10] Goering M Espinoza CN Mercier A Eason EK Johnson CW Richter CG. Moral identity in relation to emotional well-being: a meta-analysis. Front Psychol. (2024) 15:1346732. doi: 10.3389/fpsyg.2024.1346732 39118847 PMC11307125

[11] Reynolds-Tylus T Harvell-Bowman LA Sarlo ME. The impact of mortality salience on organ donation attitude, beliefs, and behavior. J Health Commun. (2021) 26:76–82. doi: 10.1080/10810730.2021.1891484 33657983

[12] Bolt S Venbrux E Eisinga R Kuks JB Veening JG Gerrits PO. Motivation for body donation to science: more than an altruistic act. Ann Anat. (2010) 192:70–4. doi: 10.1016/j.aanat.2010.02.002 20188530

[13] Sachdeva S Iliev R Medin DL. Sinning saints and saintly sinners: the paradox of moral self-regulation. Psychol Sci. (2009) 20:523–8. doi: 10.1111/j.1467-9280.2009.02326.x 19320857

[14] Malti T Krettenauer T. The relation of moral emotion attributions to prosocial and antisocial behavior: a meta-analysis. Child Dev. (2013) 84:397–412. doi: 10.1111/j.1467-8624.2012.01851.x 23005580

[15] He Y Liu Q Wen H Yuan L Luo C Yuan R . The relationship between body donation and attitudes toward death and altruism in Changsha City in China. Anat Sci Educ. (2023) 16:1200–8. doi: 10.1002/ase.2309 37328430

[16] Chen WL. Body donation registration in Taiwan: reasons and associated psychological factors. Healthcare. (2023) 11:969. doi: 10.3390/healthcare11070969 37046896 PMC10093931

[17] Farsides T Smith CF Sparks P. Beyond “altruism motivates body donation”. Death Stud. (2021) 6:1–9. doi: 10.1080/07481187.2021.2006827 34871142

[18] Ganapathy A Joy P Rout S Gaikwad M. The attitude of medical students toward voluntary body donation: a single institute survey and narrative review of global trends. Cureus. (2023) 15:e40775. doi: 10.7759/cureus.40775 37485180 PMC10362406

[19] Asante EA Maalman RS Ali MA Donkor YO Korpisah JK. Perception and attitude of medical students towards cadaveric dissection in anatomical science education. Ethiop J Health Sci. (2021) 31:867–74. doi: 10.4314/ejhs.v31i4.22 34703187 PMC8512940

[20] Gong R Xu N Lin R Gu Y Xia Y Yang W . The influence of systemic and regional anatomy on medical students' body donation willingness and the underlying factors. Anat Sci Int. (2026) 101:305–13. doi: 10.1007/s12565-025-00857-y 40542939

[21] Qin J Zhou L Zhang Y Chen Q Chen Y. The anchor of traditional culture and medical students' knowledge, attitudes, and willingness towards organ donation during internships: a cross-sectional descriptive study. Transplant Proc. (2024) 56:1222–30. doi: 10.1016/j.transproceed.2024.05.033 38991902

[22] Yang H. A cross-cultural adaptation and application of the Death Anxiety Scale (doctoral dissertation). Beijing University of Chinese Medicine, Beijing (2011).

[23] Liu N Cao Y Xu H. Prosocial behavior associated with trait mindfulness, psychological capital and moral identity among medical students: a moderated mediation model. Front Psychol. (2024) 15:1431861. doi: 10.3389/fpsyg.2024.1431861 39635702 PMC11614594

[24] Rai P Kapoor K. Receiving a donation call to dissection tables: various aspects of whole-body donation in northern union territory region medical college of India. Anat Cell Biol. (2024) 57:238–45. doi: 10.5115/acb.23.211 38575558 PMC11184417

[25] Harmon-Jones E Simon L Greenberg J Pyszczynski T Solomon S McGregor H. Terror management theory and self-esteem: evidence that increased self-esteem reduces mortality salience effects. J Pers Soc Psychol. (1997) 72:24–36. doi: 10.1037/0022-3514.72.1.24 9008372

[26] Ryan RM Deci EL. Self-determination theory and the facilitation of intrinsic motivation, social development, and well-being. Am Psychol. (2000) 55:68–78. doi: 10.1037/0003-066X.55.1.68 11392867

[27] Pyszczynski T Greenberg J Solomon S. A dual-process model of defense against conscious and unconscious death-related thoughts: an extension of terror management theory. Psychol Rev. (1999) 106:835–45. doi: 10.1037/0033-295X.106.4.835 10560330

[28] Li TY Mao YH Liang XY Jiang CM Sun HY. Death reflection vs. death anxiety: divergent paths to pro-sociality. Death Stud. (2025) 27:1–12. doi: 10.1080/07481187.2025.2607432 41454893

[29] Jin SV Ryu E. “The greedy I that gives”—The paradox of egocentrism and altruism: terror management and system justification perspectives on the interrelationship between mortality salience and charitable donations amid the COVID-19 pandemic. J Consum Aff. (2022) 56:414–48. doi: 10.1111/joca.12381 34226753 PMC8242723

[30] Zhao Y Miao Q Qiu L Hu T. A qualitative study of the spirituality of volunteers registered for human organ donation. BMC Med Ethics. (2025) 26:17. doi: 10.1186/s12910-025-01173-w 39891138 PMC11783770

[31] Gotowiec S van Mastrigt S. Having versus doing: the roles of moral identity internalization and symbolization for prosocial behaviors. J Soc Psychol. (2019) 159:75–91. doi: 10.1080/00224545.2018.1454394 29565781

[32] Benner P. Curricular and pedagogical implications for the Carnegie Study, educating nurses: a call for radical transformation. Asian Nurs Res. (2015) 9:1–6. doi: 10.1016/j.anr.2015.02.001 25829203

[33] Benner P. Educating nurses: a call for radical transformation—how far have we come? J Nurs Educ. (2012) 51:183–4. doi: 10.3928/01484834-20120402-01 22476535

[34] Garrino L Contratto C Massariello P Dimonte V. Caring for dying patient and their families: the lived experiences of nursing students in Italy. J Palliat Care. (2017) 32:127–33. doi: 10.1177/0825859717745169 29187085

[35] Yuan R Liu Q Luo C Ding S He Y. Death anxiety, sense of meaning in life, and willingness to body donation among nursing students in anatomy course: an explanatory sequential mixed study. Nurse Educ Today. (2024) 137:106160. doi: 10.1016/j.nedt.2024.106160 38503248

[36] Horiuchi-Hirose M Fukuoka T Saito S. Learning outcomes of nursing students' experience with cadaveric dissection: a scoping review. SAGE Open Nurs. (2024) 10:23779608241274543. doi: 10.1177/23779608241274543 39169993 PMC11337174

[37] Shapiro J. Walking a mile in their patients' shoes: empathy and othering in medical students' education. Philos Ethics Humanit Med. (2008) 3:10. doi: 10.1186/1747-5341-3-10 18336719 PMC2278157

[38] Champney TH Hildebrandt S Gareth Jones D Winkelmann A. BODIES R US ethical views on the commercialization of the dead in medical education and research. Anat Sci Educ. (2019) 12:317–25. doi: 10.1002/ase.1809 30240149

[39] Meiring KO Gibbon VE Alblas A. Anatomical human body donation in South Africa: inconsistencies of informed consent. Ann Anat. (2024) 255:152292. doi: 10.1016/j.aanat.2024.152292 38906340

[40] Riederer BM. Plastination and its importance in teaching anatomy: critical points for long-term preservation of human tissue. J Anat. (2014) 224:309–15. doi: 10.1111/joa.12056 23621482 PMC3931543

[41] Sedikides C Gebauer JE. Religiosity as self-enhancement: a meta-analysis of the relation between socially desirable responding and religiosity. Pers Soc Psychol Rev. (2010) 14:17–36. doi: 10.1177/1088868309351002 19940283

[42] Vail KE III Juhl J Arndt J Vess M Routledge C Rutjens BT. When death is good for life: considering the positive trajectories of terror management. Pers Soc Psychol Rev. (2012) 16:303–29. doi: 10.1177/1088868312440046 22490977

[43] Li Y Dong W Tang H Guo X Wu S Lu G . Correlates of death anxiety for patients with cancer: a systematic review and meta-analysis. J Clin Nurs. (2024) 33:1933–47. doi: 10.1111/jocn.17021 38284499

[44] Martela F Ryan RM. The benefits of benevolence: basic psychological needs, beneficence, and the enhancement of well-being. J Pers. (2016) 84:750–64. doi: 10.1111/jopy.12215 26249135

[45] Pittman J Gerstl B Cavazzoni E Rogers NM Lotz M Deans R. Deceased donor uterus transplantation: religious perceptions. Front Transplant. (2025) 4:1536754. doi: 10.3389/frtra.2025.1536754 40094108 PMC11906711

[46] White KM Starfelt Sutton LC Zhao X. Charitable donations and the theory of planned behaviour: a systematic review and meta-analysis. PLoS ONE (2023) 18:e0286053. doi: 10.1371/journal.pone.0286053 37205662 PMC10198540

