# Supplementary material for: Moral identity internalization and symbolization in relation to body donation willingness: a cross-sectional study among 2,327 Chinese health professions students
Source: Front Public Health. 2026 Oct 8;14:1929251. doi: 10.3389/fpubh.2026.1929251 (PMC13687326; doi:10.3389/fpubh.2026.1929251)
Supplement: Supplementary file 1 [file Data_Sheet_1.pdf]

A: Templer's Death Anxiety Scale (T-DAS) - English and Chinese Versions

A：坦普勒死亡焦虑量表（T-DAS）中英文版本

Item No.	English Version	Chinese Version
1	I am very much afraid of dying.	我非常害怕死亡。
2*	I rarely think about death.	我很少想到死亡。
3*	It doesn't make me nervous when people talk about death.	人们谈论死时我不会紧张。
4	I dread to think that I will have to have an operation.	我想到自己要接受手术治疗会害怕。
5*	I am not at all afraid of dying.	我一点也不害怕死亡。
6*	I am not particularly afraid of getting cancer.	我不是很害怕患癌症。
7*	The thought of death never bothers me.	我从来不会因想到死而烦恼。
8	I often think about how short life really is.	我常常为时间过得飞快而痛苦。
9	I fear dying a painful death.	我害怕痛苦地死去。
10	The subject of life after death troubles me greatly.	关于死后的话题令我非常困扰。
11	I am really scared of having a heart attack.	我很害怕心脏病发作。
12	I often think about how short life is.	我经常会想生命如此短暂。
13	I shudder when I hear people talk about World War III.	当听到人们谈论世界末日时，我会吓得发抖。
14	The sight of a dead body is horrifying to me.	我看到尸体会毛骨悚然。
15*	I feel that the future holds nothing for me to fear.	我对未来没有什么可恐惧的。

Note: Items marked with * are reverse-scored. Forward-scored items (1, 4, 8, 9, 10, 11, 12, 13, 14): "Yes" = 1 point, "No" = 0 points. Reverse-scored items (2, 3, 5, 6, 7, 15): "Yes" = 0 points, "No" = 1 point. Total score range: 0-15 points, with higher scores indicating greater death anxiety. The English version provided here is a translation of the Chinese adaptation by Yang et al. [22], which was itself developed from the original scale by Templer [3]. The Chinese version used in this study is the adaptation by Yang et al. [22].

B: The Moral Identity Scale (MIS) - English and Chinese Versions
B：道德认同量表（MIS）中英文版本

Instructions:

Listed below are nine characteristics that may describe a person: caring, compassionate, fair, friendly, generous, helpful, hardworking, honest, and kind. Please imagine these characteristics as part of who you are. For a moment, visualize in your mind what kind of person you would be with these characteristics. Imagine how you would think, feel, and act. When you have a clear image of yourself with these characteristics, please answer the following questions.

Item No.	English Version	Chinese Version
**Moral Identity Scale internalization (MIS-Internalization)**		
1	It would make me feel good to be a person who has these characteristics.	成为具有这些特征的人会让我感到满足。
2	Being someone who has these characteristics is an important part of who I am.	拥有这些特征是我自我认知的重要部分。
3*	I would be ashamed to be a person who has these characteristics.	成为具有这些特征的人我会感到羞愧。
4*	Having these characteristics is not really important to me.	拥有这些特征对我来说不重要。
5	I strongly desire to have these characteristics.	我强烈渴望拥有这些特征。
**Moral Identity Scale symbolization (MIS-Symbolization)**		
6	I often wear clothes that identify me as having these characteristics.	我经常穿能表明我拥有这些特征的衣服。
7	The types of things I do in my spare time (e.g., hobbies) clearly identify me as having these characteristics.	我闲暇时所从事的活动类型（如爱好）清晰地表明我拥有这些特征。
8	The kinds of books and magazines that I read identify me as having these characteristics.	我阅读的书籍和杂志类型表明我拥有这些特征。
9	The fact that I have these characteristics is communicated to others by my membership in certain organizations.	我通过成为某些组织的成员，向他人传达我拥有这些特质。
10	I am actively involved in activities that communicate to others that I have these characteristics.	我积极参与能向他人表达我拥有这些特征的活动。

Note: Items marked with * are reverse-scored. Forward-scored items (1, 2, 5, 6, 7, 8, 9, 10): 1 = Strongly Disagree to 5 = Strongly Agree. Reverse-scored items (3, 4): 5 = Strongly Disagree to 1 = Strongly Agree. Total score range: 10-50 points, with higher scores indicating stronger moral identity. MIS-Internalization items: 1, 2, 3, 4, 5. MIS-Symbolization items: 6, 7, 8, 9, 10.

The Chinese version was developed by the present authors following standard translation-back-translation procedures, as described in the Methods section.
